# Supplementary material for: MiR-302d inhibits TGFB-induced EMT and promotes MET in primary human RPE cells
Source: PLoS One. 2022 Nov 28;17(11):e0278158. doi: 10.1371/journal.pone.0278158 (PMC9704570; doi:10.1371/journal.pone.0278158)
Supplement: S1 Raw images — (PDF) [file pone.0278158.s001.pdf]

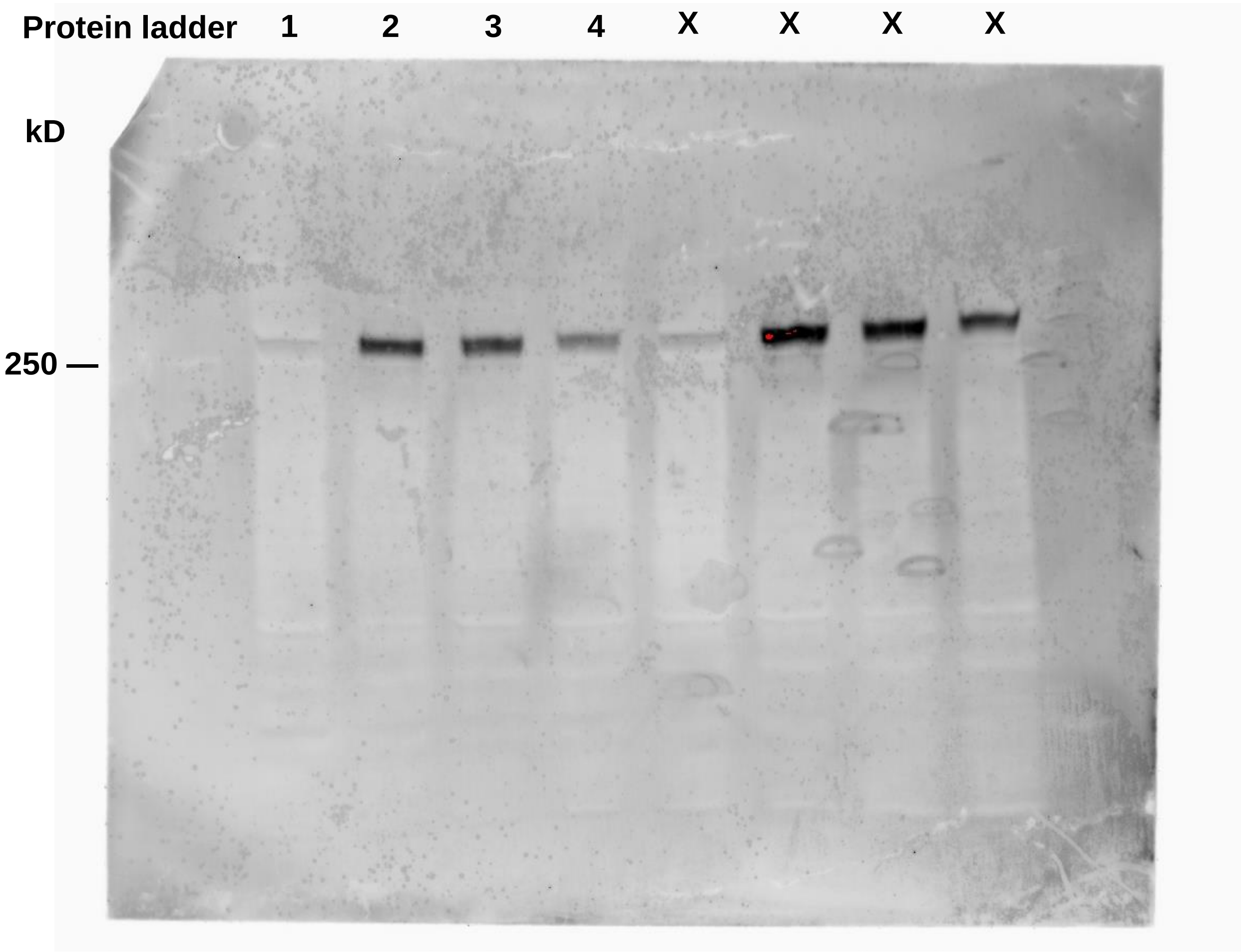

Raw blot image of FN1 for Fig 4B. 1: without TGFB1 exposure, 2: mock transfection + TGFB1, 3: NC transfection + TGFB1, 4: miR-302d transfection + TGFB1 (the other lanes are unrelated samples). The image was captured with ChemiDoc MP Imaging System (Bio-Rad).

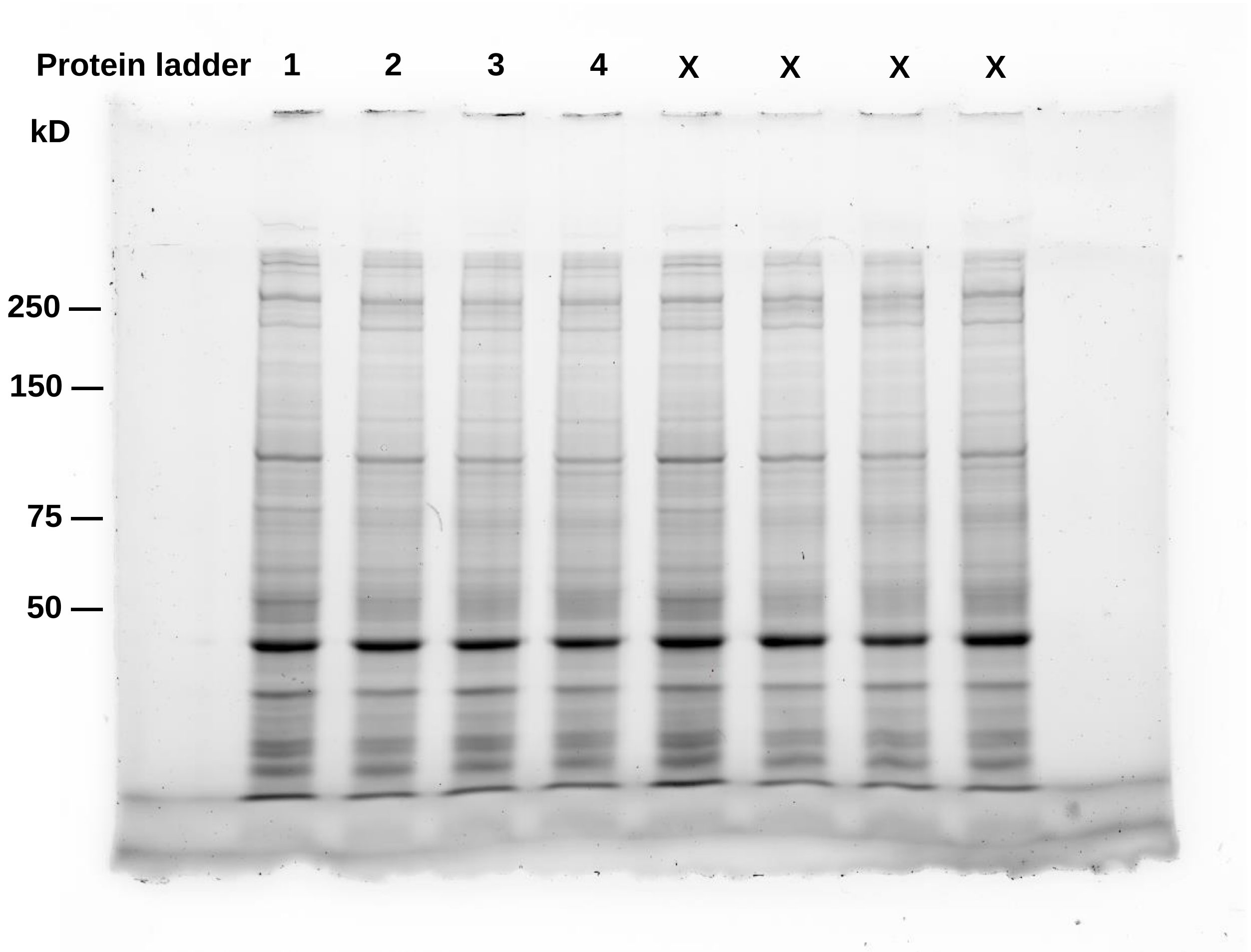

Raw stain-free blot image for Fig 4B. 1: without TGFB1 exposure, 2: mock transfection + TGFB1, 3: NC transfection + TGFB1, 4: miR-302d transfection + TGFB1 (the other lanes are unrelated samples). The image was captured with ChemiDoc MP Imaging System (Bio-Rad).

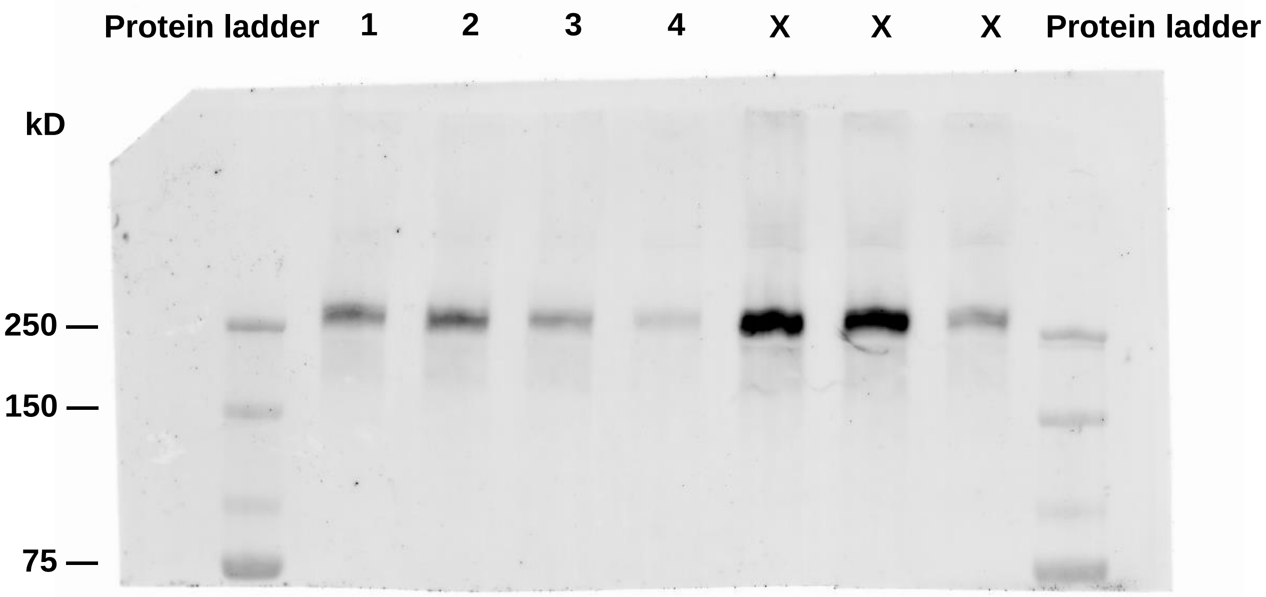

Raw blot image of FN1 for Fig 8B. 1: TGFB1 + mock transfection, 2: TGFB1 + NC transfection, 3: TGFB1 + miR-302d transfection, 4: TGFB1 + SB431542 (the other lanes are unrelated samples). The image was captured with ChemiDoc MP Imaging System (Bio-Rad).

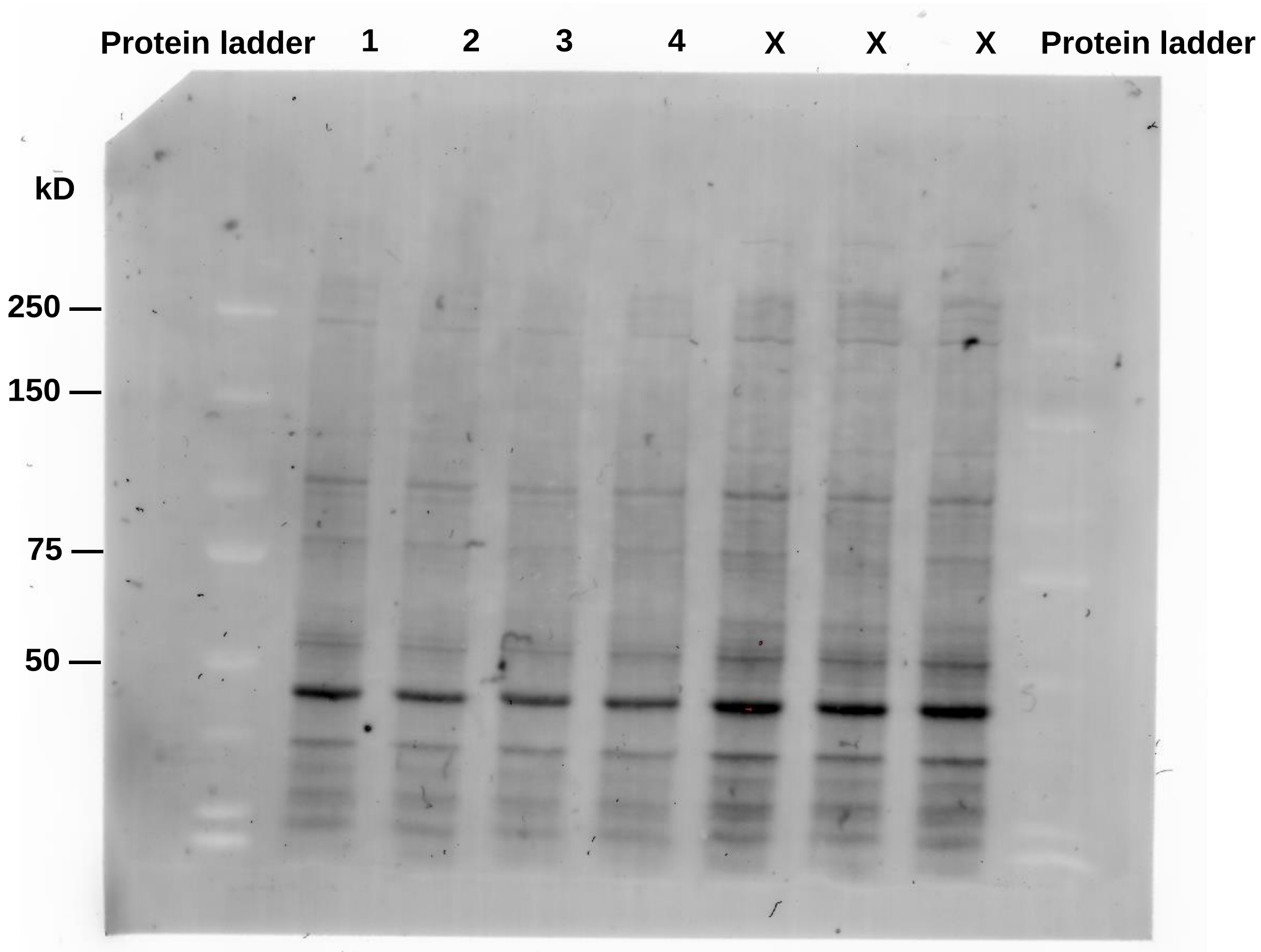

Raw stain-free blot image for Fig 8B. 1: TGFB1 + mock transfection, 2: TGFB1 + NC transfection, 3: TGFB1 + miR-302d transfection, 4: TGFB1 + SB431542 (the other lanes are unrelated samples). The image was captured with ChemiDoc MP Imaging System (Bio-Rad).

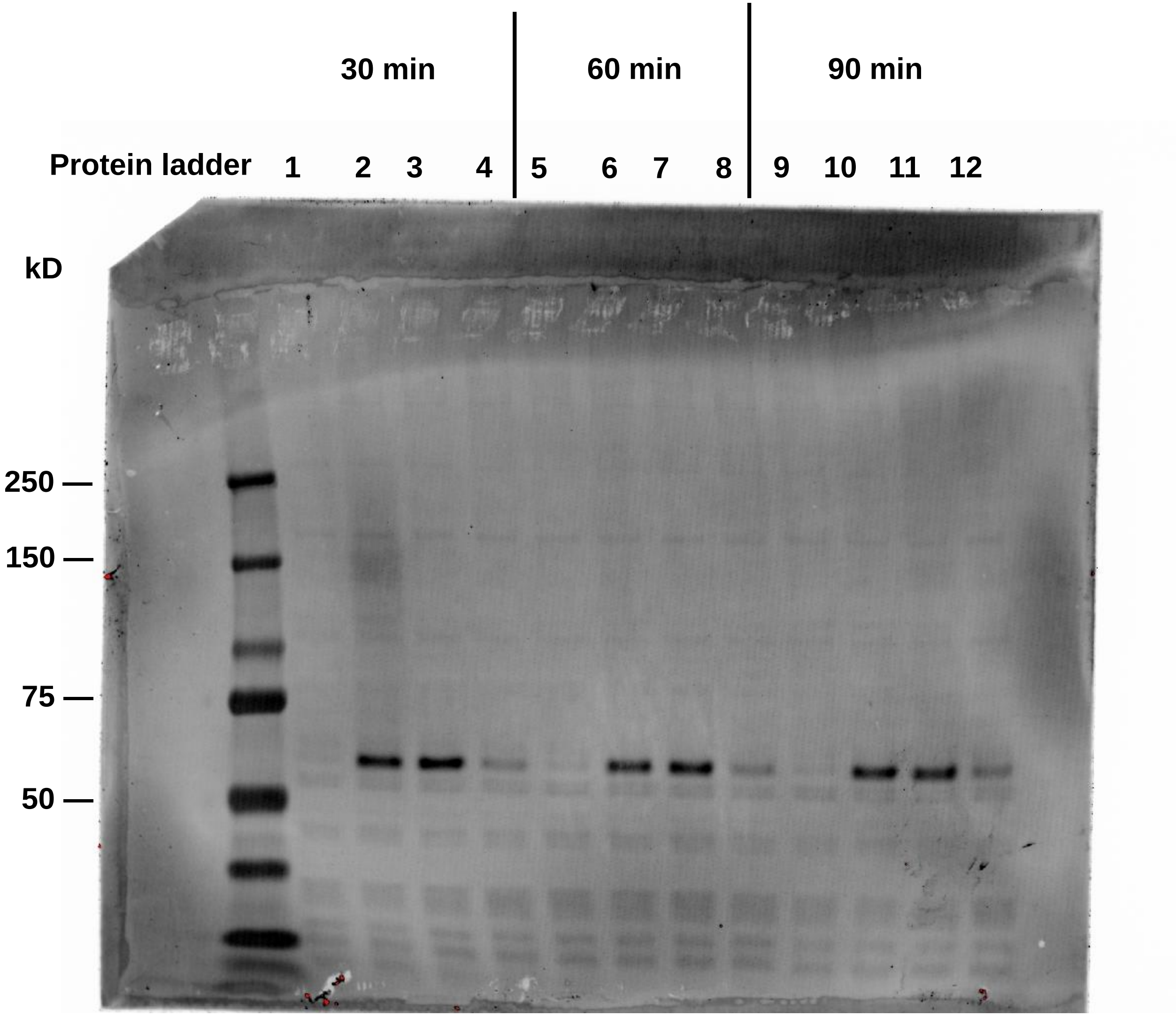

Raw blot image of pSMAD2 for Fig 9B, S1B Fig, and S2B Fig. 1, 5, 9: without TGFB1 exposure, 2, 6, 10: mock transfection + TGFB1, 3, 7, 11: NC transfection + TGFB1, 4, 8, 12: miR-302d transfection + TGFB1. The image was captured with ChemiDoc MP Imaging System (Bio-Rad).

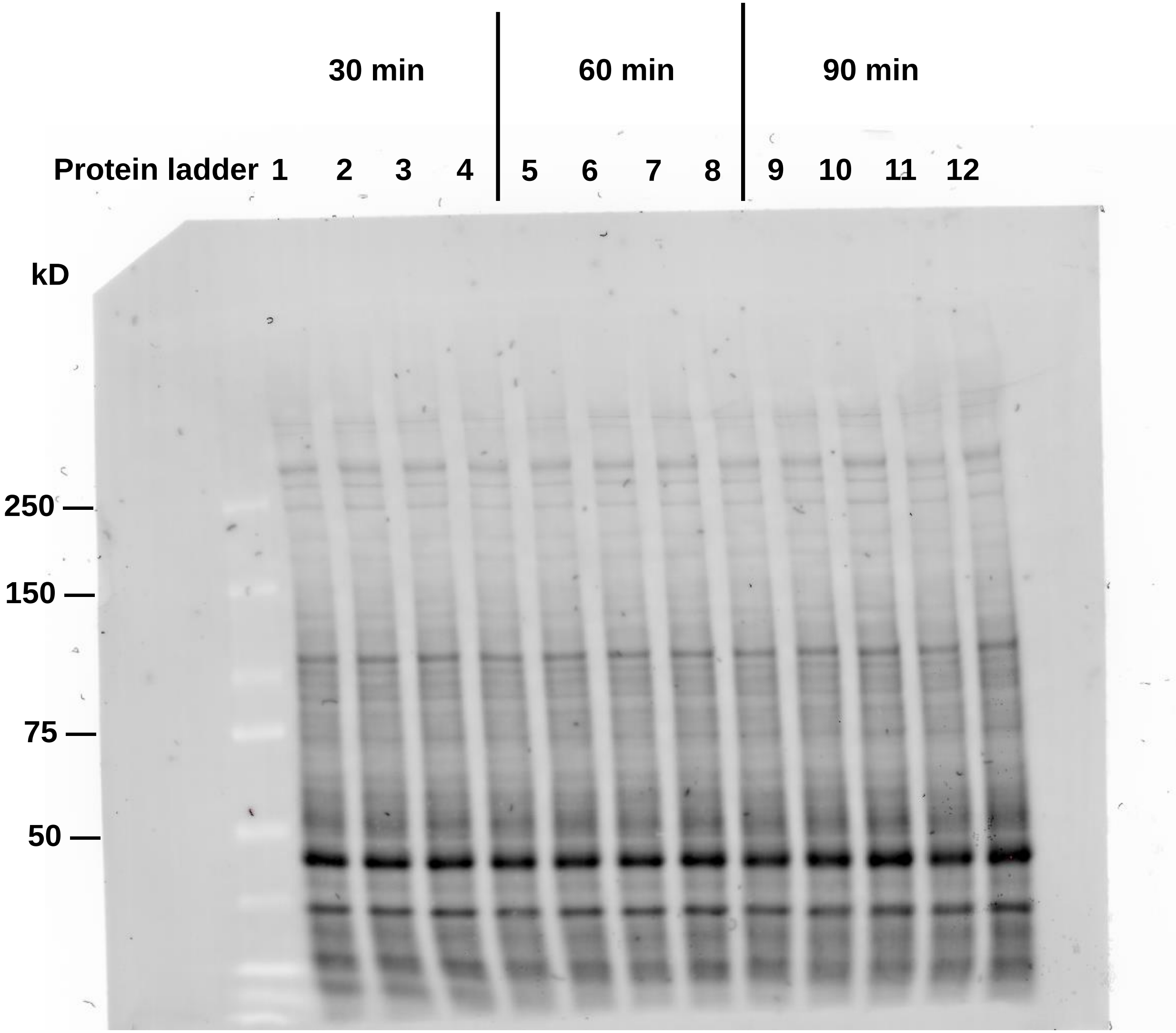

Raw stain-free blot image for Fig 9B, S1B Fig, and S2B Fig. 1, 5, 9: without TGFB1 exposure, 2, 6, 10: mock transfection + TGFB1, 3, 7, 11: NC transfection + TGFB1, 4, 8, 12: miR-302d transfection + TGFB1. The image was captured with ChemiDoc MP Imaging System (Bio-Rad).
