# Supplementary figures and images for: MiR-302d inhibits TGFB-induced EMT and promotes MET in primary human RPE cells
Source: PLoS One. 2022 Nov 28;17(11):e0278158. doi: 10.1371/journal.pone.0278158 (PMC9704570; doi:10.1371/journal.pone.0278158)

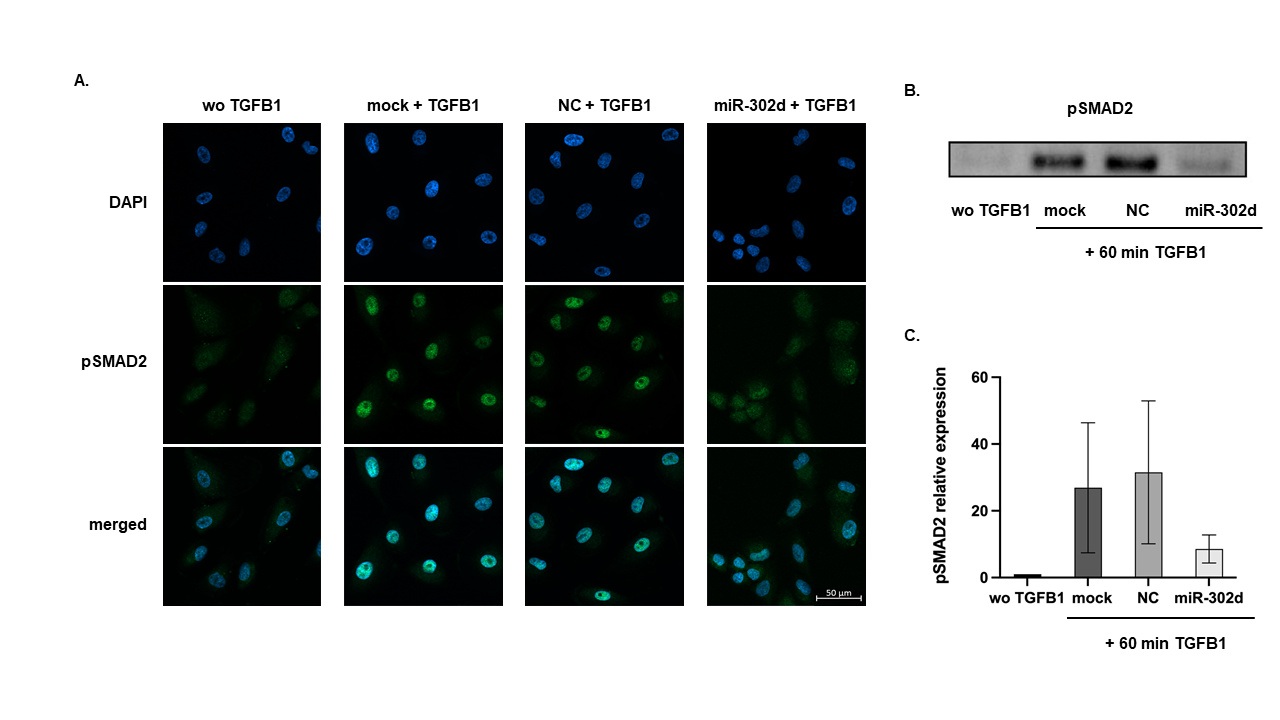

Supplement: S1 Fig — hRPE cells were treated with or without TGFB1 for 60 min 24 h after transfection with mock, NC, or miR-302d. (A) DAPI (blue) and pSMAD2 (green) staining in hRPE cells. The scale bar represents 50 μm. (B) Representative Western blot of pSMAD2 60 min after initial TGFB1 exposure. (C) The relative expression of pSMAD2 normalized to total proteins (S1 Raw images). The values were further normalized to the "wo TGFB1" group. (TIF) [file pone.0278158.s002.TIF]

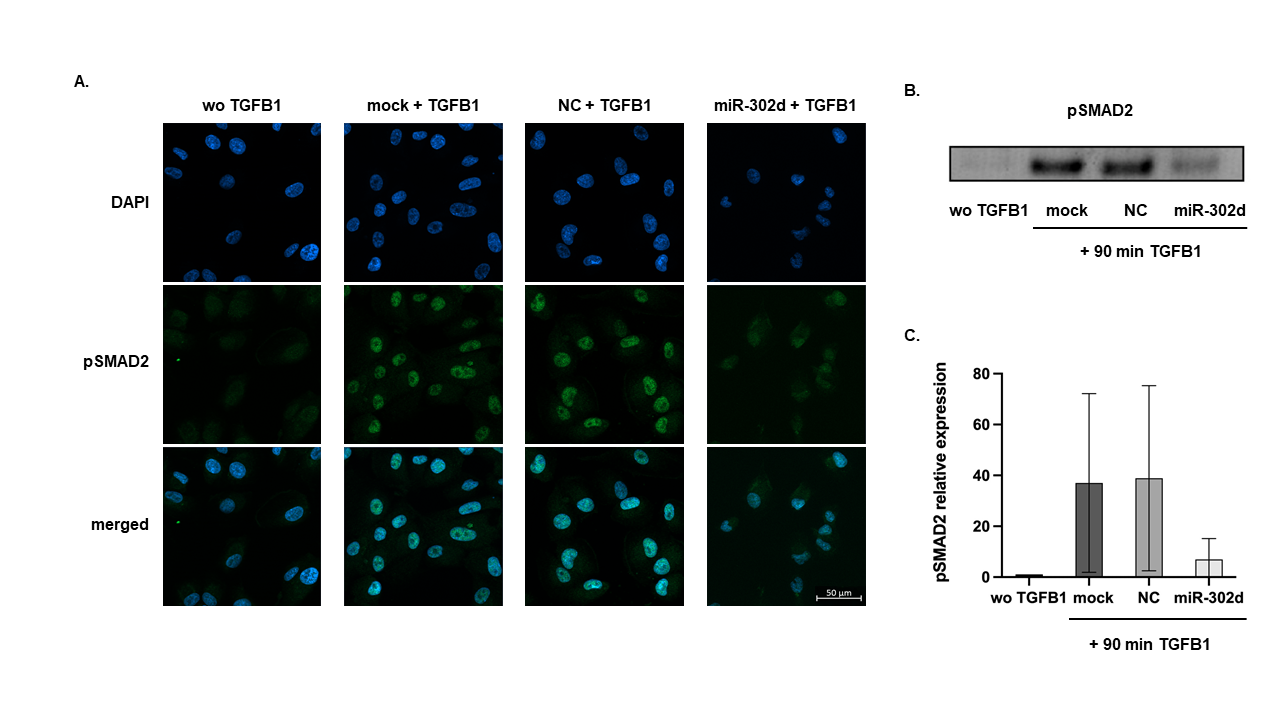

Supplement: S2 Fig — hRPE cells were treated with or without TGFB1 for 90 min 24 h after transfection with mock, NC, or miR-302d. (A) DAPI (blue) and pSMAD2 (green) staining in hRPE cells. The scale bar represents 50 μm. (B) Representative Western blot of pSMAD2 90 min after initial TGFB1 exposure. (C) The relative expression of pSMAD2 normalized to total proteins (S1 Raw images). The values were further normalized to the "wo TGFB1" group. (TIF) [file pone.0278158.s003.TIF]
